# Commensal bacteria at the crossroad between cholesterol homeostasis and chronic inflammation in atherosclerosis[S]

**DOI:** 10.1194/jlr.M072165

**Published:** 2017-02-27

**Authors:** Kazuyuki Kasahara, Takeshi Tanoue, Tomoya Yamashita, Keiko Yodoi, Takuya Matsumoto, Takuo Emoto, Taiji Mizoguchi, Tomohiro Hayashi, Naoki Kitano, Naoto Sasaki, Koji Atarashi, Kenya Honda, Ken-ichi Hirata

**Affiliations:** Division of Cardiovascular Medicine,* Department of Internal Medicine, Kobe University Graduate School of Medicine, Kobe, Japan; Department of Bacteriology,† University of Wisconsin-Madison, Madison, WI; RIKEN Center for Integrative Medical Sciences (IMS),§ Yokohama, Japan; AMED-CREST,** Japan Agency for Medical Research and Development, Tokyo, Japan

**Keywords:** cholesterol/metabolism, bile acid metabolism, nuclear receptors/farnesoid X receptor, macrophages, gut microbiota

## Abstract

The gut microbiota were shown to play critical roles in the development of atherosclerosis, but the detailed mechanism is limited. The purpose of this study is to clarify the influence of gut microbiota on atherogenesis via lipid metabolism and systemic inflammation. Germ-free or conventionally raised (Conv) ApoE-deficient (*ApoE*^−/−^) mice were fed chow diet and euthanized at 20 weeks of age. We found that the lack of gut microbiota in *ApoE*^−/−^ mice caused a significant increase in the plasma and hepatic cholesterol levels compared with Conv *ApoE*^−/−^ mice. The absence of gut microbiota changed the bile acid composition in the ileum, which was associated with activation of the enterohepatic fibroblast growth factor 15, fibroblast growth factor receptor 4 axis, and reduction of cholesterol 7α-hydroxylase and hepatic bile acid synthesis, resulting in the accumulation of liver cholesterol content. However, we found that the lack of microbiota caused a significant reduction in atherosclerotic lesion formation compared with Conv *ApoE*^−/−^ mice, which might be associated with the attenuation of lipopolysaccharide-mediated inflammatory responses. Our findings indicated that the gut microbiota affected both hypercholesterolemia and atherogenesis in mice.

There are approximately trillions of microorganisms that live in our gut. These bacteria, namely the gut microbiota, have recently attracted considerable attention. They establish a dynamic bacterial ecosystem encoding at least 150-fold more genes than the human genome (1). In addition to intestinal diseases (2), the gut microbiota are involved in cardiometabolic disorders, such as obesity (3), type 2 diabetes (4), and nonalcoholic fatty liver disease (5, 6). The gut microbiota are also associated with the development and progression of atherosclerotic cardiovascular diseases (7, 8). Although both chronic inflammation and dyslipidemia are recognized as important contributors to atherosclerosis, knowledge on the potential role of the microbiota in regulating chronic inflammation and cholesterol homeostasis during atherosclerotic plaque growth is limited.

Metabolic endotoxemia is known to be a low-grade elevation in plasma lipopolysaccharide (LPS) and is associated with obesity. Recent studies also show that metabolic endotoxemia participates in chronic inflammation and the development of cardiometabolic diseases, such as diabetes and atherosclerosis (9). LPS is the major molecular component of the outer membrane of Gram-negative bacteria that comprise about 70% of the total bacteria in the gut (10). LPS contains lipid A that is a pathogen-associated molecular pattern, which activates pro-inflammatory pathways upon binding to its pattern recognition receptor, Toll-like receptor 4 (TLR4) (11). TLR4 expresses on the cell surface of monocytes, macrophages, and various other cell types (11). High-fat diet increases gut permeability and enhances the penetration of gut microbiota-derived endotoxins into the circulation resulting in metabolic endotoxemia (12). Meanwhile, germ-free (GF) mice are resistant to high-fat diet-induced insulin resistance and obesity (13). Although associations with gut dysbiosis and various chronic diseases have been observed, it remains to be clarified whether microbiota-derived LPS results in the development of atherosclerosis.

Over the past years, S. L. Hazen and colleagues (7, 14) have splendidly reported that the gut microbial-derived metabolites, trimethylamine and trimethylamine N-oxide, are pro-atherogenic in both mice and humans and their levels in circulation are strongly linked to cardiovascular disease risk. Although there are some studies using GF ApoE-deficient (*ApoE*^−/−^) mice, the role of gut microbiota on atherogenesis and its mechanism remain largely unknown (15, 16). Considering the fact that atherosclerosis is one of chronic inflammatory diseases and that dysbiosis causes low-grade inflammation both systemically, through increased leakage of bacterial products such as LPS, and locally in the intestine (17); there is speculation that the gut microbiota may regulate atherogenesis through modulation of the immune system as well as trimethylamine N-oxide. Moreover, lipidomic analysis on GF and conventionally raised (Conv) mice proposed that the commensal bacteria affect host lipid metabolism (18). A detailed mechanism for how the gut microbiota may contribute to host lipid metabolism could potentially be explained by microbial regulation of lipoprotein lipase activity. Despite a key role of cholesterol balance in atherosclerotic cardiovascular diseases, only a few studies have shown the potential role of the microbiota in regulating whole-body cholesterol homeostasis (19).

Bile acids are synthesized from cholesterol in the liver via the classic pathway initiated by cholesterol 7α-hydroxylase (CYP7A1) and are excreted into the intestine. In the intestine, bile acids restrict bacterial proliferation and overgrowth, whereas bacterial enzymes modify primary bile acid through deconjugation, dehydrogenation, dehydroxylation, and sulfation reactions to produce secondary bile acids. About 95% of the bile acids are reabsorbed in the distal ileum and returned to the liver via the enterohepatic cycle. This enterohepatic circulation of bile acids is maintained via a negative feedback control of their synthesis (20). The remaining 5% of bile acids are eliminated in the feces. Hepatic conversion of cholesterol to bile acid balances fecal excretion, and this process is the major route for cholesterol catabolism (21). Indeed, bile acids participate in the regulation of dietary lipid absorption and act as signaling molecules, modulating lipid metabolism and energy homeostasis (22). We hypothesized that modified bile acid component in the presence of gut microbiota would affect the cholesterol metabolism and the development of atherosclerosis.

The purpose of this study is to clarify the influence of gut microbiota on the development of atherosclerotic lesion formation, systemic immune responses, and cholesterol and bile acid homeostasis using Conv and GF atherosclerosis-prone *ApoE*^−/−^ mice fed a chow diet. Here, we show that the absence of microbiota inhibits atherosclerotic lesion formation in the aortic sinuses, consistent with decreased plasma LPS levels and inflammatory cytokines in aortas and macrophages. Furthermore, we observed that GF *ApoE*^−/−^ mice have altered cholesterol and bile acid homeostasis, identifying gut microbiota as an effective therapeutic target for treating atherosclerosis and cardiovascular diseases.

## MATERIALS AND METHODS

### Animals and experimental design

We used *ApoE*^−/−^ mice on the C57BL/6 background, originally provided by Prof. Shun Ishibashi (Department of Endocrinology and Metabolism, Jichi Medical School, Japan). To obtain GF *ApoE*^−/−^ mice, specific pathogen-free female *ApoE*^−/−^ mice underwent hysterectomy at the end of pregnancy. The newborns (F1 generation) were delivered by Cesarean section after washing the uterus in antiseptic solution, and were transferred to a GF isolator. GF C57B6/J female mice were used as their foster mothers, and further colonies of GF *ApoE*^−/−^ mice were maintained in the isolator. Female GF (F2 generation or later) or Conv *ApoE*^−/−^ mice were fed a chow diet composed of 20% calories from fat, 50% calories from carbohydrate, and 30% calories from protein, sterilized with 50 kGy γ irradiation (CMF; Oriental Yeast Co., Ltd., Tokyo, Japan; supplemental Table S1) and were housed in the same facility except that GF mice were maintained in vinyl isolators. We confirmed that there was no contamination in the isolators throughout the experiment. All mice were bred at RIKEN Yokohama Institute, shipped to Kobe University on the day of euthanization, and euthanized at 20 weeks of age. All mice were not fasted to keep them GF because they were in sterile transport cages with food and water. All animal experiments were approved by the Institutional Animal Care and Use Committee (permission number: P120707) and conducted in accordance with the Guidelines for Animal Experiments at Kobe University School of Medicine, RIKEN Yokohama Institute, and University of Wisconsin-Madison, as well as the Public Health Service guidelines.

### Analysis of lipid profile and bile acids in serum and liver

Blood samples were drawn by cardiac puncture under anesthesia using 2,2,2-tribromoethanol (250 mg/kg intraperitoneal injection; Wako Pure Chemical Industries, Osaka, Japan). Plasma was acquired by centrifugation and stored at −30°C until measurement. The total cholesterol, LDL, HDL, and triglyceride contents were measured with an automated chemistry analyzer. In addition, 50 μl plasma were used for lipoprotein profiling by high-performance liquid chromatography using molecular sieve columns with the LipoSEARCH system (Skylight Biotech Inc., Tokyo, Japan) (23). For determination of hepatic triglyceride and cholesterol concentrations, total lipids were extracted from the liver by the Folch method (24), and the hepatic triglyceride and cholesterol contents were measured using the enzyme assay. Total bile acids in the liver were measured with a total bile acid test (Wako Pure Chemical Industries, Osaka, Japan).

### Atherosclerotic lesion assessments

Atherosclerotic lesions were assessed as previously described (25). Briefly, mice were anesthetized and the aorta was perfused with saline. To assess the atherosclerotic lesion size at the aortic sinus, the samples were cut in the ascending aorta, and the proximal samples containing the aortic sinus were embedded in OCT compounds (Tissue-Tek; Sakura Finetek, Tokyo, Japan). Five consecutive sections (10 μm thickness) taken at 100 μm intervals (i.e., 150, 250, 350, 450, and 550 μm from the bottom of the aortic sinus) were collected from each mouse and stained with Oil Red O (Wako Pure Chemical Industries). Plaque area, vessel area, and Oil Red O-positive area were measured using ImageJ (National Institutes of Health, Bethesda, MD). The volume of atherosclerosis in the aortic sinus was expressed as the mean size of the five sections for each mouse. The fraction of plaque area to vessel area was also calculated, which allowed us correction for errors due to angulated sections that might lead to overestimations of the plaque area. Immunohistochemistry was performed on formalin-fixed cryosections (10 μm) of aortic roots using antibodies to identify macrophages (MOMA-2, 1:400; BMA Biomedicals, Augst, Switzerland) and CD4^+^ T cells (CD4, clone H129.19, 1:100; BD Biosciences, San Jose, CA), followed by detection with biotinylated secondary antibodies and streptavidin-HRP. Stained sections were observed under an all-in-one type fluorescence microscope (BZ-8000; Keyence, Osaka, Japan) using the BZ Analyzer software (Keyence). Stained sections were digitally captured, and the stained area was calculated. Quantitative analyses of CD4^+^ T cells in the atherosclerotic lesion were performed by counting the positive-stained cells and dividing by total plaque area.

### Analysis of bile acids in fecal samples and intestinal contents

Bile acids were extracted from fecal samples and intestinal contents using a method previously described (26) with small modifications. The fecal sample (100 mg) was put in a 2 ml tube with zirconia beads, suspended in 900 μl of 50 mM cold sodium acetate buffer (pH 5.6)/ethanol mixture (1:3, v/v), vortexed, and heated at 80°C for 30 min. The sample was vortexed at 5 m/s for 45 s using FastPrep 24 (MP Biomedicals) and centrifuged at 1,300 *g* for 10 min. The supernatant (200 μl) was mixed with 800 μl of MilliQ in a 2 ml tube. The sample was applied to a Bond Elut C_18_ cartridge (500 mg/6 ml, Agilent Technologies; Santa Clara, CA). The cartridge was washed with 10% ethanol (5 ml) and then bile acids were eluted with ethanol (5 ml). The solvent was evaporated and the residue was dissolved in 1 ml of ethanol. The solution was diluted with 50% ethanol and transferred to a vial after filtration using a 0.2 μm filter (Millex-LG; Millipore, Billerica, MA). Quantification of bile acids was performed on a Waters Acquity UPLC system with an Acquity UPLC BEH C_18_ column (2.1 × 150 mm, pore size 1.7 μm; Waters, Milford, MA) coupled with Waters Xevo G2-S QTOF mass spectrometer with an electrospray ionization probe. Injection volume was 5 μl. Mobile phase A was water and mobile phase B was acetonitrile, both containing 0.1% formic acid. The flow rate was 0.4 ml/min. The autosampler temperatures were kept at 60°C and 10°C, respectively. The Waters Xevo G2-S QTOF was run in negative mode (scan 50–850 amu at a rate of 0.3 scans per second). The following instrument conditions were used: capillary, 0.5 kV; source temperature, 150°C; sampling cone, 20 V; cone gas, 100 l/h; desolvation gas flow, 1,000 l/h at 450°C. To ensure mass accuracy and reproducibility, leucine enkephalin was used as the reference lock-mass (*m/z* 554.2615) with a lock-mass spray. Data analysis was performed by TargetLynx software (Waters).

### Plasma LPS analysis

The plasma LPS concentration was determined by Limulus amebocyte lysate assay (Lonza, Switzerland) according to the manufacturer’s instructions. We used pyrogen-free glass tubes (Lonza) for LPS measurements.

### Preparation of peritoneal macrophages

GF or Conv *ApoE*^−/−^ mice were treated with 3% thioglycollate broth intraperitoneal injection and euthanized by rapid cervical dislocation for peritoneal macrophage isolation after a 3 day treatment, as described previously (25). We confirmed that there was no endotoxin in the thioglycollate broth. Cells were plated onto culture dishes with RPMI medium containing 10% FBS and incubated for 3–4 h at 37°C and 5% CO_2_. The adhesive cells were used as macrophages for quantitative PCR analysis.

### Real-time RT-PCR analysis

Total RNA was extracted from liver, ileum, peritoneal macrophages, and whole aorta after perfusion with RNAlater (Ambion, Austin, TX) using TRIzol reagent (Invitrogen, Carlsbad, CA). For RT, a PrimeScript RT reagent kit (Takara, Shiga, Japan) was used. Quantitative PCR was performed using a SYBER Premix Ex Taq (Takara) and a LightCycler 96 system (Roche Diagnostics, Mannheim, Germany) according to the comparative threshold cycle method following the manufacturer’s protocol. The primers used are shown in supplemental Table S2.

### Ileal FGF15 protein

Fibroblast growth factor 15 (FGF15) concentrations were measured from ileal tissue extracts using a commercial ELISA kit (USCN Life Science; kit number SEL154Mu). ELISA was done according to the manufacture’s protocol.

### CYP7A1 enzyme activity assay

Mouse liver microsomes were isolated for the analysis of CYP7A1 enzyme activity with a high-performance liquid chromatography-based method, as described previously (27).

### Cytokine analysis

Plasma cytokines and chemokines were analyzed by Mouse Cytokine Array, Panel A (R&D Systems, Minneapolis, MN). A total of 40 cytokines/chemokines were analyzed in this assay, which was performed according to the manufacturer’s protocol. Briefly, aliquots of 15 μl cytokine array detection antibody cocktail were added to each test sample and incubated at room temperature for 1 h. Cytokine array membranes were then incubated with sample/antibody mixtures overnight at 4°C on a shaker. Test samples on membranes were washed three times with 1× wash buffer, each for 10 min. Membranes were then incubated with streptavidin-HRP solution for 30 min at room temperature on a shaker. Membranes were washed three times again, as before. An aliquot of 1 ml Chemi reagent mix was added onto each membrane and incubated for 1 min. The membrane was drained and exposed to a CCD imager for 1 min. Data were analyzed using ImageJ software. Mean values of spotted duplicates were calculated and normalized with the mean values of reference spots (i.e., internal positive controls). For ELISA assay, plasma cytokines were analyzed for interleukin (IL)-6 and TNF-α using paired antibodies specific for corresponding cytokines according to the manufacturer’s instructions (R&D Systems).

### Statistical analysis

Data are expressed as the mean ± SEM. The Mann-Whitney U test was used to detect significant differences between two groups. A value of *P* < 0.05 was considered statistically significant. For statistical analysis, GraphPad Prism version 6.0 (GraphPad Software, San Diego, CA) was used.

## RESULTS

### The gut microbiota influence plasma lipid levels in *ApoE* KO mice

**Fig. 1.**
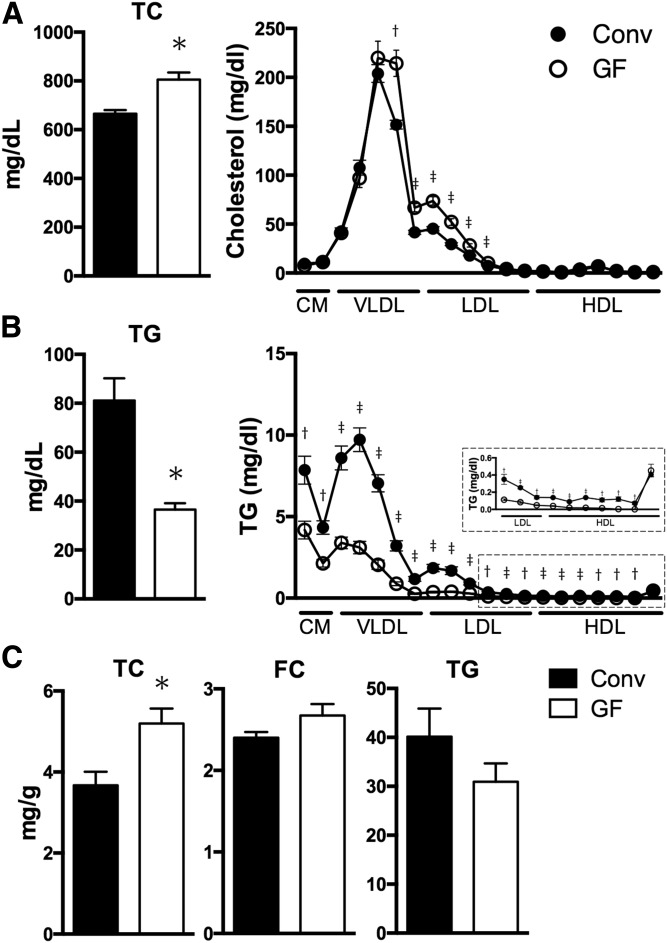
Effects of the lack of microbiota on cholesterol and triglyceride metabolism in *ApoE*^−/−^ mice. A, B: Blood samples from Conv (n = 7) and GF (n = 7) *ApoE*^−/−^ mice were prepared for plasma total cholesterol (A, left) and triglycerides (TG) (B, left). Fresh plasma from Conv (n = 5) and GF (n = 5) *ApoE*^−/−^ mice was analyzed for lipoprotein profiles of cholesterol (A, right) and triglycerides (B, right) by high-performance liquid chromatography. An enlarged figure (inset) was added in the TG lipoprotein profile. C: The graphs represent the hepatic cholesterol and triglyceride in Conv (n = 4) and GF (n = 4) *ApoE*^−/−^ mice. Mean values ± SEM are plotted; *P < 0.05, ^†^*P* <0 .01, ^‡^*P* < 0.001 versus Conv, Mann-Whitney U test. CM, chylomicron; TC, total cholesterol; FC, free cholesterol.

It has been reported that levels of serum cholesterol in WT GF mice are comparable to those in WT specific pathogen-free mice (28). To clarify whether the microbial deletion affects plasma cholesterol and triglyceride levels in hypercholesterolemic *ApoE*^−/−^ mice, we assessed the lipid profile using an automated chemistry analyzer. Unexpectedly, GF *ApoE*^−/−^ mice showed a significant increase in plasma total cholesterol and LDL cholesterol levels and a decrease in triglyceride levels compared with Conv mice (**Fig. 1A, B**, **Table 1**). We further classified and quantified plasma lipoproteins by high-performance liquid chromatography. Analysis of plasma lipoprotein fractions revealed a pronounced elevation of cholesterol in the small VLDL and LDL fractions in GF *ApoE*^−/−^ mice, whereas triglyceride content was lower in the chylomicron and triglyceride-rich VLDL fraction (Fig. 1A, B). Although the presence of gut microbiota did not affect liver free cholesterol levels, we also noted increased levels of liver total cholesterol in GF *ApoE*^−/−^ mice (Fig. 1C). A trend toward a decrease in liver triglyceride was observed in GF *ApoE*^−/−^ mice, but did not reach statistical significance (Fig. 1C). Taken together, these results indicate that gut microbiota could regulate cholesterol and triglyceride homeostasis in *ApoE*^−/−^ mice.

**TABLE 1.** Body weight and plasma lipid profile in Conv and GF *ApoE*^−/−^ mice

	Conv	GF
Body weight (g)	23.4 ± 0.8	23.6 ± 0.7
HDL-cholesterol (mg/dl)	14.3 ± 1.2	14.3 ± 1.2
LDL-cholesterol (mg/dl)	103.4 ± 4.7	192.0 ± 6.2*^a^*

Results are expressed as mean ± SEM. Conv (n = 7) and GF (n = 7) *ApoE*^−/−^ mice are used.

a *P* < 0.05 versus Conv.

### The gut microbiota regulate bile acid homeostasis in *ApoE* KO mice

**Fig. 2.**
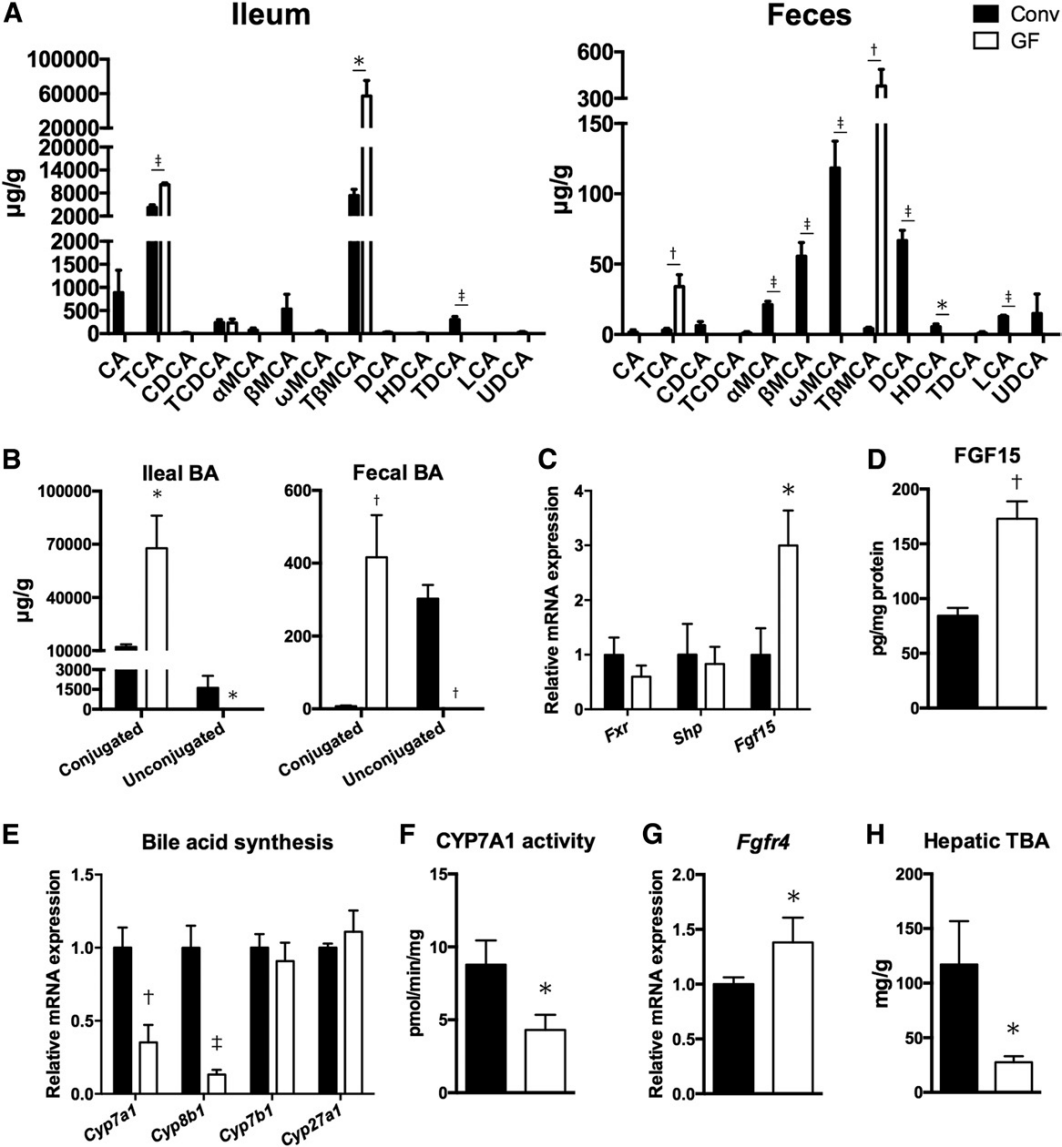
Microbial regulation of intestinal and hepatic genes involved in bile acid homeostasis. A: UPLC-Q-TOF/MS analyses of bile acids in the distal ileum and feces from Conv (n = 5) and GF (n = 5) *ApoE*^−/−^ mice. B: Conjugated and unconjugated bile acids in the distal ileum and feces from Conv (n = 5) and GF (n = 5) *ApoE*^−/−^ mice. C: Expression of genes involved in FXR signaling in the distal ileum from Conv (n = 4) and GF (n = 5) *ApoE*^−/−^ mice. D: Ileal FGF15 protein from Conv (n = 5) and GF (n = 5) *ApoE*^−/−^ mice. E, G: The graphs represent gene expression of enzymes and a molecule involved in bile acid biosynthesis in the liver from Conv (n = 5) and GF (n = 5) *ApoE*^−/−^ mice. F: Hepatic CYP7A1 activity from Conv (n = 5) and GF (n = 5) *ApoE*^−/−^ mice. H: Total bile acids in the liver from Conv (n = 4) and GF (n = 4) *ApoE*^−/−^ mice. Mean values ± SEM are plotted; *P < 0.05, ^†^*P* < 0.01, ^‡^*P* < 0.001 versus Conv, Mann-Whitney U test. CA, cholic acid; CDCA, chenodeoxycholic acid; DCA, deoxycholic acid; GCA, glycocholic acid; GCDCA, glycochenodeoxycholic acid; GDCA, glycodeoxycholic acid; HDCA, hyodeoxycholic acid; LCA, lithocholic acid; UDCA, ursodeoxycholic acid; T, taurine-conjugated; BA, bile acid; TBA, total bile acids.

To assess how the gut microbiota regulate lipid metabolism, we focused on the bile acids because they are synthesized from cholesterol in the liver, then excreted into the intestine, and converted into secondary bile acids by the gut microbiota. UPLC-Q-TOF/MS-based analysis of bile acids in the distal ileum and feces from GF and Conv *ApoE*^−/−^ mice revealed that the absence of gut microbiota affected the composition of bile acids both in the small intestine and the feces (**Fig. 2A**). The distal ileum from GF mice exclusively contained taurocholic acid (TCA) and tauro-β-muricholic acid (TβMCA), whereas that from Conv mice included a variety of bile acids other than TCA and TβMCA, for example, cholic acid and β-muricholic acid (MCA), indicating that the gut microbiota would effectively deconjugate taurine-conjugated bile acids in the small intestine. Considering that the fecal bile acids in Conv mice were dominated by ωMCA, βMCA, and deoxycholic acid, it was assumed that further microbial modifications of bile acids would also occur in the colon of Conv mice. We confirmed that most of the fecal bile acids in Conv mice were unconjugated bile acids, whereas all bile acids in both ileum and feces from GF mice were conjugated (Fig. 2B). Next, we investigated gene expressions involved in bile acid transporters in the distal ileum and found that the expressions of apical bile acid transporter [multidrug resistance-associated protein 2 (*Mrp2*)] and basolateral transporter [organ solute transporter α (*Osta*)] were upregulated in the distal ileum of GF mice, while the absence of gut microbiota had less effect on other apical bile acid transporters [ileal bile acid transporter (*Ibat*), ileal bile acid binding protein (*Ibabp*)] and basolateral transporters (*Mrp3*, *Ostb*) (supplemental Fig. S1). This result also might be attributed to the altered composition of bile acids because the uptake of conjugated bile acids is mediated by active transport, whereas unconjugated bile acids are absorbed by passive diffusion across the apical brush border membrane (29). Farnesoid X receptor (FXR) is a member of the nuclear receptor superfamily and has emerged as a key player in the control of multiple metabolic pathways, including bile acid homeostasis (30). We therefore investigated to determine whether altered bile acid composition in GF mice influences FXR signaling in the ileum. Although the absence of gut microbiota had less effect on expressions of *Fxr* and small heterodimer partner (*Shp*), the expression of *Fgf15*, a molecular target of FXR, was significantly upregulated in the distal ileum of GF mice (Fig. 2C). Ileal FGF15 protein was also increased in GF mice (Fig. 2D). Taken together, the gut microbiota regulate the composition of bile acids and the activity of FXR signaling in the ileum of *ApoE*^−/−^ mice.

Considering that bile acids undergo the enterohepatic circulation, we determined whether the altered bile acid composition in GF *ApoE*^−/−^ mice was associated with microbial regulation of hepatic enzymes in bile acid synthesis. The mRNA expressions of enzymes involved in bile acid synthesis, *Cyp7a1* and *Cyp8b1*, were significantly downregulated in the liver of GF mice (Fig. 2E). In particular, CYP7A1, the rate-limiting enzyme for the classical pathway of bile acid biosynthesis, plays an important role in regulation of bile acid and cholesterol homeostasis. We confirmed that hepatic CYP7A1 activity is also reduced in GF mice (Fig. 2F). FGF15 produced in the distal small intestine was shown to bind fibroblast growth factor receptor 4 (FGFR4) in hepatocytes and inhibit the expression of the CYP7A1 gene (31). To clarify the mechanism of suppressed bile acid synthesis in GF *ApoE*^−/−^ mice, expression of *Fgfr4* in the liver was analyzed. Surprisingly, we found that expression of *Fgfr4* was significantly upregulated in the liver of GF mice (Fig. 2G) due to increased FGF15 in the ileum (Fig. 2C, D), which was consistent with the previous report (32). In concordance with reduced bile acid synthesis, analysis of bile acids revealed that the absence of gut microbiota reduced the content of total bile acids in the liver (Fig. 2H). Collectively, activation of the FGF15-FGFR4 axis was associated with the suppression of bile acid synthesis via inhibition of CYP7A1 in GF *ApoE*^−/−^ mice.

### The gut microbiota affect cholesterol and fatty acid synthesis in the liver

**Fig. 3.**
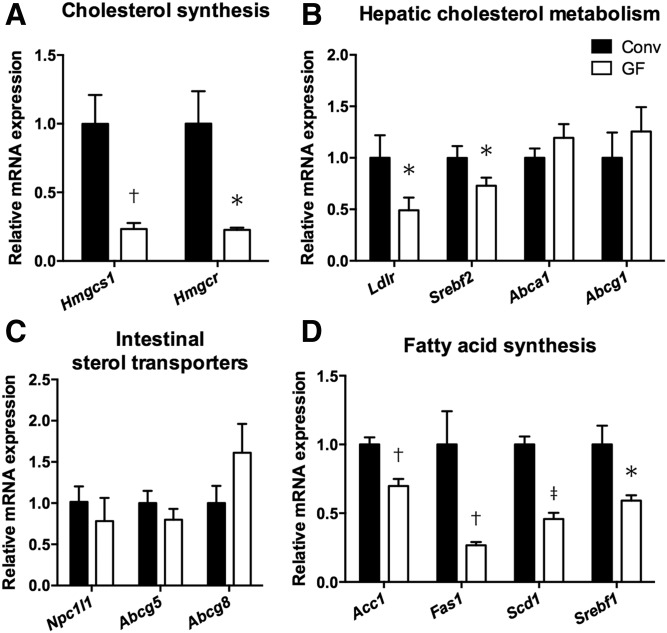
Bacterial regulation of hepatic and intestinal genes involved in cholesterol homeostasis. A–D: The graphs represent gene expression of enzymes and molecules involved in cholesterol synthesis (A), cholesterol metabolism (B), and fatty acid synthesis (D) in the liver, as well as cholesterol transport in the small intestine (C) from Conv (n = 4) and GF (n = 5) *ApoE*^−/−^ mice. Mean values ± SEM are plotted; *P < 0.05, ^†^*P* < 0.01, ^‡^*P* < 0.001 versus Conv, Mann-Whitney U test.

Next, we determined whether the altered bile acid profile in GF mice was associated with microbial regulation of enzymes in the cholesterol homeostasis. We found that expressions of genes involved in cholesterol synthesis, HMG-CoA synthase 1 (*Hmgcs1*) and HMG-CoA reductase (*Hmgcr*), were downregulated in the liver of GF mice (**Fig. 3A**). Therefore, it was likely that cholesterol synthesis was suppressed due to a negative feedback for excessive cholesterol in the liver of GF mice. The expression of *Ldlr* in the liver of GF mice was significantly suppressed compared with that of Conv mice (Fig. 3B), which might lead to the increase of plasma LDL levels. The LDLR gene is regulated by SREBP-2, also known as sterol regulatory element-binding transcription factor 2 (SREBF-2), and we found that the expression of *Srebf2* was also downregulated in GF mice (Fig. 3B). There was no differential expression of *Abca1* and *Abcg1* between Conv and GF mice. Although bile acids could affect cholesterol transport in the intestine (33), the altered bile acid composition had less effect on the expression of genes related to intestinal cholesterol transport, Niemann-Pick C1-like 1 (*Npc1l1*), *Abcg5*, and *Abcg8* in the ileum (Fig. 3C). Expressions of genes involved in fatty acid synthesis in the liver, acetyl-CoA carboxylase (*Acc1*), *Fas1*, stearoyl-CoA desaturase 1 (*Scd1*), and *Srebf1*, were downregulated in GF mice (Fig. 3D), suggesting that endogenous fatty acid synthesis was also suppressed in the absence of gut microbiota. Thus, lack of gut microbiota altered bile acid profile, which might lead to the stimulation of the enterohepatic FGF15/FGFR4 axis, the reduction of hepatic *Cyp7a1* expression, bile acid synthesis, cholesterol catabolism, and, consequently, the increase of hepatic cholesterol levels. Further, it might reduce the uptake of LDL-cholesterol to the liver and subsequently increase plasma cholesterol levels in GF mice.

### GF *ApoE* KO mice are resistant to the development of atherosclerosis

**Fig. 4.**
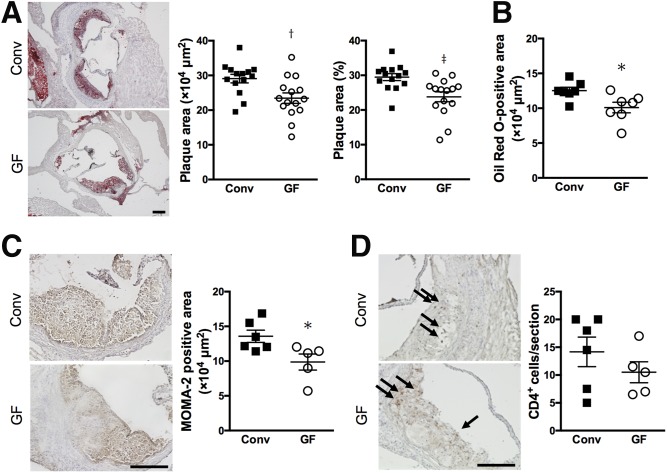
Attenuated atherosclerosis development in the absence of microbiota. A: Representative photomicrographs of Oil Red O staining in the aortic sinus from Conv (n = 14) and GF (n = 15) *ApoE*^−/−^ mice. Black bars represent 200 μm. Quantitative analysis of atherosclerotic plaque size and the fraction of plaque area to vessel area in the aortic sinus was performed. B: Quantitative analysis of intraplaque lipid area as visualized by Oil Red O staining was performed. C, D: Representative photomicrographs and quantitative analyses of MOMA-2-positive macrophages (C) and CD4^+^ T cells (D) in the aortic sinus from Conv (n = 6) and GF (n = 5) *ApoE*^−/−^ mice. Black bars represent 200 μm. Mean values ± SEM are plotted; *P < 0.05, ^†^*P* < 0.01, ^‡^*P* < 0.001 versus Conv, Mann-Whitney U test.

To determine the effects of commensal bacteria on the development of atherosclerosis, we assessed the atherosclerotic lesion formation in the aortic root. No adverse effects were observed in both groups throughout the experiment. There was no difference in body weight between Conv and GF *ApoE*^−/−^ mice (Table 1). Interestingly, lack of gut microbiota in *ApoE*^−/−^ mice fed chow diet caused a significant reduction in both atherosclerotic plaque size and the fraction of plaque area to vessel area compared with Conv mice (29.1 ± 1.2 × 10^4^ μm^2^ and 29.5 ± 1.0% in Conv *ApoE*^−/−^ mice, 23.5 ± 1.5 × 10^4^ μm^2^ and 23.8 ± 1.4% in GF *ApoE*^−/−^ mice; **Fig. 4A**). GF *ApoE*^−/−^ mice also showed a decreased Oil Red O-positive lipid area (Fig. 4B). Immunohistochemical analysis of atherosclerotic lesions revealed that GF mice showed a reduction of intraplaque macrophages (Fig. 4C), whereas the lack of microbiota did not affect CD4 T cells in the aortic sinus (Fig. 4D). Taken together, commensal bacteria influence the plaque formation and macrophage accumulation within lesions in mice.

### The gut microbiota affect systemic inflammation in *ApoE* KO mice

**Fig. 5.**
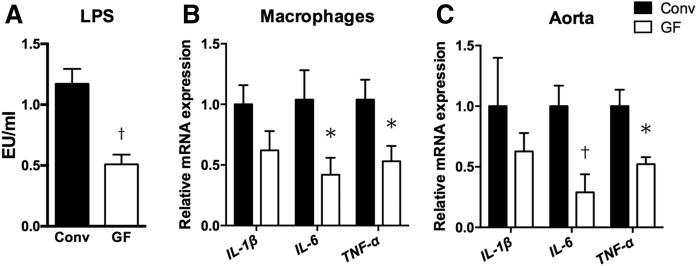
Attenuated metabolic endotoxemia in the absence of microbiota. A: LPS levels in plasma of Conv (n = 5) and GF (n = 5) *ApoE*^−/−^ mice. B, C: The graphs represent gene expression of *IL-1β*, *IL-6*, and *TNF-α* in thioglycollate-induced peritoneal macrophages (B) and the aorta (C) from Conv (n = 5) and GF (n = 5) *ApoE*^−/−^ mice. Mean values ± SEM are plotted; *P < 0.05, ^†^*P* < 0.01 versus Conv, Mann-Whitney U test.

We found that GF *ApoE*^−/−^ mice had reduced atherosclerotic lesions compared with Conv mice, despite the result that GF *ApoE*^−/−^ mice had increased plasma cholesterol levels. To reveal the mechanism of plaque reduction in GF mice, we focused on immune responses because several studies in GF animals have demonstrated that microbial populations of the gut are essential for the complete development of a normal immune response (34, 35). Plasma LPS level was decreased in GF *ApoE*^−/−^ mice compared with Conv mice (**Fig. 5A**). The LPSs detected in GF mice were likely derived from the sterile diet we used (36). Because the atherosclerotic lesions of GF *ApoE*^−/−^ mice showed a marked reduction in macrophage accumulation (Fig. 4C) and LPS could induce pro-inflammatory activity in macrophages via TLR4, we analyzed inflammatory gene expression in peritoneal macrophages. Gene expressions of *IL-6* and *TNF-α* in macrophages from GF *ApoE*^−/−^ mice were significantly reduced compared with those from Conv *ApoE*^−/−^ mice (Fig. 5B). Next, to determine the role of gut microbiota on the inflammatory cytokines in aorta, we performed real-time quantitative PCR analysis of whole aortas. We also observed that expressions of *IL-6* and *TNF-α* in aortas from GF *ApoE*^−/−^ mice were significantly reduced, while expression of *IL-1β* was not changed (Fig. 5C). Moreover, we performed cytokine array analysis with plasma samples. GF mice showed a significant reduction of pro-inflammatory adhesion molecules and chemokines, such as soluble intracellular adhesion molecule 1 (sICAM-1), C-X-C motif ligand (CXCL)-1, and CXCL-12 compared with Conv mice (supplemental Fig. S2A). Cytokine ELISA showed plasma IL-6 and TNF-α levels were also reduced in GF mice (supplemental Fig. S2B), suggesting that the absence of gut microbiota resulted in the reduction of inflammatory cytokines and chemokines in aortas as well as in blood. Collectively, our findings suggest that GF *ApoE*^−/−^ mice have immune-regulatory properties, including decreased plasma LPS levels and decreased pro-inflammatory molecules in macrophages and plasma, which may lead to the reduction of aortic inflammation and atherosclerotic lesion formation in GF *ApoE*^−/−^ mice.

## DISCUSSION

In this study, we demonstrated that GF *ApoE*^−/−^ mice are resistant to the development of atherosclerosis. In addition, we identified a profound role of gut microbiota not only on inflammatory responses, but also as regulator of hepatic bile acid synthesis and cholesterol homeostasis. Lack of microbiota reduced plasma LPS levels and pro-inflammatory cytokine gene expressions in macrophages and aorta. Accordingly, we can suggest that GF *ApoE*^−/−^ mice had less inflammatory activity and a reduced plaque burden in the aortic sinus. In contrast, we showed that the absence of gut microbiota suppressed bile acid deconjugation and diversity of bile acid composition, which might cause the reduction of hepatic cholesterol catabolism and excretion and the increase of plasma cholesterol levels in hypercholesterolemic mice. These events were associated with changes in the increment of the enterohepatic FGF15-FGFR4 axis and suppressed hepatic bile acid synthesis. Collectively, our results provided in vivo evidence that gut microbiota promote atherogenesis in the aortic sinus partially through amplifying systemic pro-inflammatory responses, whereas commensal bacteria keep plasma cholesterol at a low level via inducing hepatic bile acid biosynthesis.

We found that the absence of microbiota caused an increased abundance of primary bile acids, mainly TCA and TβMCA, and a decreased abundance of secondary bile acids in the ileum as well as in feces due to the impaired microbial deconjugation and metabolism of bile acids. Bile acids function as signaling molecules, regulate their own biosynthesis, and modulate key metabolic pathways involved in lipoprotein, glucose, and energy metabolism by activation of nuclear receptors such as FXR (37). FGF15 is downstream of ileal FXR and directly responsible for hepatic CYP7A1 suppression. It has been reported that FGF15 is transported to the liver where it binds FGFR4 and acts to suppress CYP7A1 in the liver (30). Our data indicate that the altered bile acid composition, especially the abundance of TCA and TβMCA, is associated with upregulation of the enterohepatic FGF15-FGFR4 axis. However, Sayin et al. (28) recently reported that TβMCA could be an FXR antagonist, which suppresses ileal *Fgf15* expression and enhances hepatic *Cyp7a1* expression in GF WT mice. Because ApoE plays an important role in maintaining gut homeostasis (38), there is a possibility that the ApoE^−/−^ condition might cause this discrepancy, but further studies will be needed.

In the present study, significant elevations in plasma and liver cholesterol were observed in GF *ApoE*^−/−^ mice. The increase of liver cholesterol in GF *ApoE*^−/−^ mice can be explained by decreased conversion from cholesterol to bile acids in the liver, as supported by downregulation of *Cyp7a1* and *Cyp8b1*. This was consistent with decreased total bile acids in the liver of GF *ApoE*^−/−^ mice. Previously, it has demonstrated that ApoB-containing lipoprotein cholesterol is reduced in CYP7A1 transgenic mice (39, 40), but increased in CYP7A1 KO mice (40), implying a crucial role for hepatic CYP7A1 in cholesterol homeostasis. Because the gene expression of *Hmgcr* was found to be positively correlated with its enzymatic activity (41), cholesterol biosynthesis might be decreased in the liver of GF *ApoE*^−/−^ mice. Moreover, decreased hepatic expression of cholesterologenic genes may reflect an increase in cholesterol levels in the liver of GF *ApoE*^−/−^ mice. This was supported by the downregulation of *Srebf2*, a key regulator of cholesterol homeostasis, and *Ldlr*, a SREBP-2 target gene and a major pathway by which LDL is cleared from the bloodstream. In addition to endogenous cholesterol production and excretion, it was recently suggested that interaction between dietary lipids and microbiota regulates cholesterol metabolism (42). In the current study, irradiated chow diet was used to avoid the effect of excessive dietary lipids on the gut microbiota. Although dietary cholesterol absorption can contribute to an amount of hepatic cholesterol storage, the lack of microbiota did not affect expression of the genes related to cholesterol transport in the ileum. Collectively, we have, for the first time, revealed that gut microbiota could regulate cholesterol homeostasis via bile acid metabolism under hypercholesterolemia (supplemental Fig. S3).

We showed that the lack of gut microbiota reduced serum levels of both chylomicron- and VLDL-triglycerides. Previous studies of GF and conventionalized mice revealed that the microbiota promote absorption of monosaccharides from the gut lumen, with resulting induction of de novo hepatic lipogenesis (43). Consistent with this, we found that the expressions of hepatic lipogenic genes, including SREBP-1c, were lower in GF mice than in Conv mice. Because we could not attempt to address whether lipid absorption from the gut and/or lipid clearance are affected in this study, additional studies will be needed in the future. In addition, an important role for FXR in this process (6) is supported by the findings that plasma triglyceride levels were reduced by administration of FXR agonists (44) and increased in FXR KO mice (45). Activated FXR signaling in GF *ApoE*^−/−^ mice might also lead to decreased hepatic lipogenesis and plasma triglyceride concentration.

In the current study, GF *ApoE*^−/−^ mice showed a significant decrease in atherosclerotic plaque area despite unfavorable high plasma cholesterol levels. We thought that anti-inflammatory status might result in the reduction of atherosclerosis. Metabolic endotoxemia is considered to be an initiating factor of cardiometabolic diseases (12). It was also shown that *Akkermansia muciniphila*, a mucin-degrading bacterium, attenuates atherosclerosis lesions by ameliorating metabolic endotoxemia-induced inflammation (46). We found that GF mice have a reduction of the circulating LPS level, which is associated with the decreased gene expressions of inflammatory cytokines, such as IL-6 and TNF-α, in macrophages and aorta. The stimulation of TLR4 with LPS induces the release of critical pro-inflammatory cytokines, and TLR4 is expressed in various vascular cells, including macrophages (11). Although we could not analyze the effect of gut microbiota on TLR4 signaling in macrophages, we found that GF mice have a significant reduction of pro-inflammatory cytokines in macrophages and aorta, suggesting that the absence of gut microbiota resulted in the reduction of inflammatory cytokines and chemokines in systemic circulation, which might result in the reduction of atherosclerosis.

In summary, our study clarified that the gut microbiota regulate the development of atherosclerosis in the aortic sinus partially through modulating not only systemic inflammation, but also cholesterol metabolism. So far, two articles have been published in which the role of microbiota in atherogenesis using GF *ApoE*^−/−^ mice has been investigated. Wright et al. (15) demonstrated that GF *ApoE*^−/−^ mice had reduced atherosclerosis after 22 weeks of high-fat feeding. The other study showed that GF mice on a chow diet developed larger lesions compared with conventionalized mice with eleven defined bacteria, although this difference was ameliorated in mice fed a Western diet (16). We compared atherosclerotic lesion size between GF and conventionally raised mice with a complex community of commensal microbes, and revealed that the gut microbiota could accelerate the formation of atherosclerosis. It is known that some bacterial species, such as probiotics, have athero-protective effects (47) and other species may worsen atherosclerosis via producing trimethylamine (7). In the future, to identify the role of each gut bacterial species on atherogenesis based on our present study could lead to a promising therapeutic avenue in the treatment of cardiovascular diseases.

## Supplementary Material

Supplemental Data
