# Supplementary material for: Commensal bacteria at the crossroad between cholesterol homeostasis and chronic inflammation in atherosclerosis
Source: J Lipid Res. 2017 Feb 27;58(3):519–28. doi: 10.1194/jlr.M072165 (PMC5335582; doi:10.1194/jlr.M072165)
Supplement: Supplemental Data [file supp_58_3_519__index.html]

Commensal Bacteria at the Crossroad between Cholesterol Homeostasis and Chronic Inflammation in Atherosclerosis — Commensal bacteria at the crossroad between cholesterol homeostasis and chronic inflammation in atherosclerosis — Supplemental Data 

# Commensal bacteria at the crossroad between cholesterol homeostasis and chronic inflammation in atherosclerosis

## Supplemental Data

- Supplementary Figures and Tables (.pdf, 565 KB) - Supplementary Figures and Tables
