# Supplementary material for: Commensal bacteria at the crossroad between cholesterol homeostasis and chronic inflammation in atherosclerosis
Source: J Lipid Res. 2017 Feb 27;58(3):519–28. doi: 10.1194/jlr.M072165 (PMC5335582; doi:10.1194/jlr.M072165)
Supplement: Supplemental Data [file 10.1194_M072165_jlr.M072165-1.pdf]

Supplementary Information

Commensal Bacteria at the Crossroad Between Cholesterol Homeostasis and Chronic Inflammation in Atherosclerosis

Kazuyuki Kasahara[1,2], Takeshi Tanoue[3], Tomoya Yamashita[1,*],

Keiko Yodoi[1], Takuya Matsumoto[1], Takuo Emoto[1], Taiji Mizoguchi[1], Tomohiro Hayashi[1], Naoki Kitano[1],

Naoto Sasaki[1], Koji Atarashi[3], Kenya Honda[3, 4], Ken-ichi Hirata[1]

[1]Division of Cardiovascular Medicine, Department of Internal Medicine, Kobe University Graduate School of Medicine, Kobe, Japan

[2]Department of Bacteriology, University of Wisconsin-Madison, Madison, USA

[3]RIKEN Center for Integrative Medical Sciences (IMS), Yokohama, Japan

[4]AMED-CREST, Japan Agency for Medical Research and Development, Tokyo, Japan

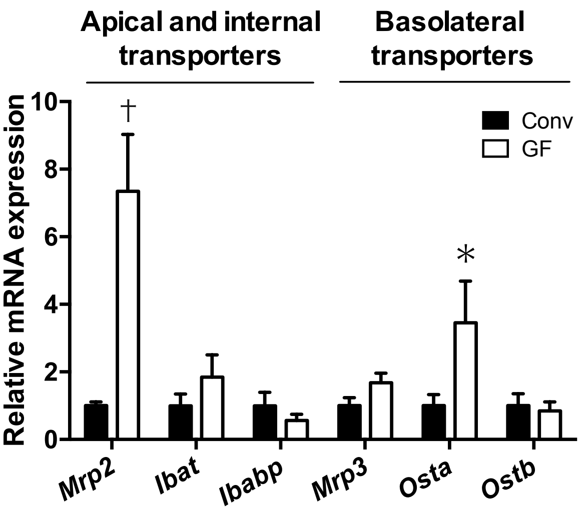

Supplementary Figure 1. Expression of genes involved in bile acid transporters in the distal ileum from Conv (n=4) and GF (n=5) *ApoE*$^{-/-}$ mice. Mean values ± SEM are plotted; *P<0.05, $^{\dagger}P$<0.01 versus Conv, Mann-Whitney *U* test. Conv, conventionally raised; GF, germ-free.

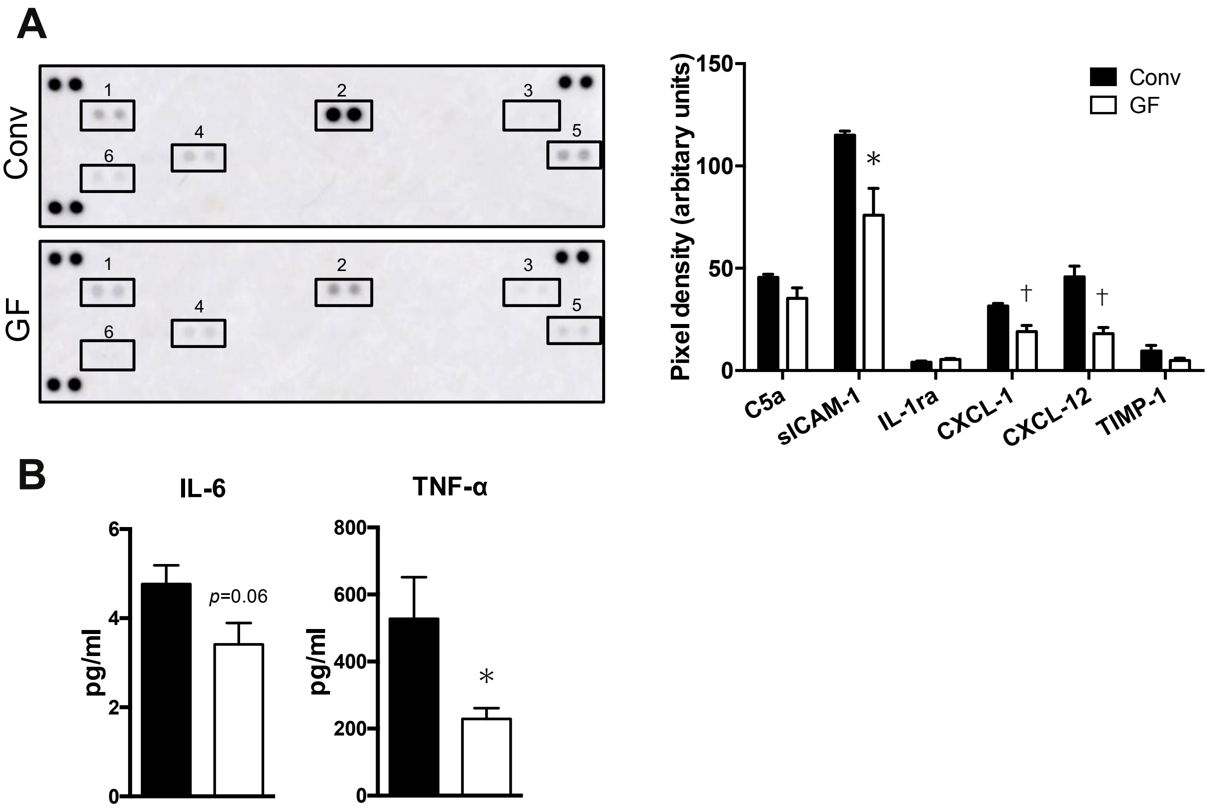

Supplementary Figure 2. (A-B) Cytokine profile in plasma of Conv and GF *ApoE*$^{-/-}$ mice. (A) The cytokine content in unstimulated plasma from Conv (n=4) and GF (n=4) *ApoE*$^{-/-}$ mice was analyzed using the cytokine array kit. Spot intensities were normalized to background and positive controls set to 100% intensity. Presented numbers on membranes mark targets as follows: 1; C5a, 2; sICAM-1, 3; IL-1ra, 4; CXCL-1, 5; CXCL-12, 6; TIMP-1. (B) Plasma IL-6 and TNF-α levels were examined by ELISA. n=5 per group. Mean values ± SEM are plotted; **P*<0.05, †*P*<0.01 versus Conv, Mann-Whitney *U* test. C5a, Complement component 5a; sICAM-1, soluble Intracellular Adhesion Molecule 1; IL-1ra, Interleukin 1 receptor antagonist; CXCL, C-X-C motif ligand; TIMP-1, Tissue Inhibitor of Metalloproteinase 1.

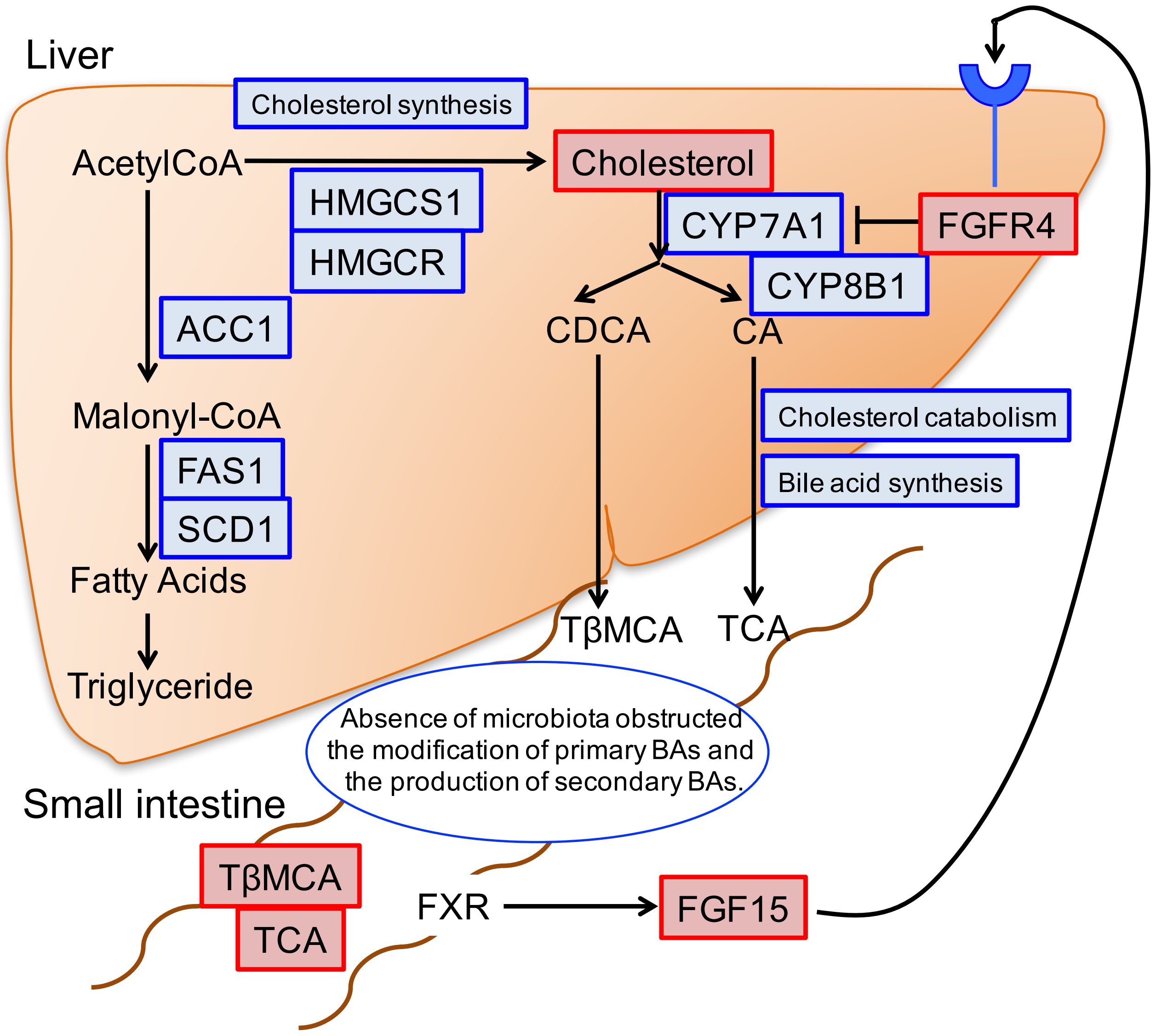

Supplementary Figure 3. Proposed model for the role of gut microbiota in the regulation of lipid metabolism. Absence of gut microbiota obstructed deconjugation and production of secondary bile acids, which might lead to the accumulation of TCA and TβMCA in the distal ileum. Altered bile acid composition, especially the increase of conjugated bile acids in the ileum, was associated with increased activity of entero-hepatic FGF15-FGFR4 axis and suppressed hepatic bile acid synthesis. The

accumulation of liver cholesterol in GF *ApoE*$^{-/-}$ mice could be explained by decreased conversion from cholesterol to bile acids in the liver, whereas the cholesterol synthesis in the liver was reduced. Lack of microbiota could result in the reduction of hepatic lipogenesis and plasma TG concentration. Red columns mean an increase or an up-regulation, and blue columns mean a reduction or a down-regulation in GF *ApoE*$^{-/-}$ mice compared to Conv mice. CA, cholic acid; CDCA, chenodeoxycholic acid; TCA, taurine-conjugated cholic acid; TβMCA, taurine-conjugated beta muricholic acid.

Supplementary Table 1.

The composition of the chow diet used in the study (CMF; Oriental Yeast Co.)

Component	Composition (g/kg)
Water	79
Protein	278
Lipids	83
Minerals	64
Dietary fibre	31
Nitrogen free extract	465
Calcium	118
Phophorus	8.3
Iron	0.2
Vitamin E	0.23
Vitamin B_1	0.04
Vitamin B_6	0.01
Niacin	0.13
Choline	2.8
Folate	0.002

Supplementary Table 2. Primers for RT-PCR

Primer Name	Sequence
Gapdh-F	TGTGTCCGTCGTGGATCTGA
Gapdh-R	TTGCTGTTGAAGTCGCAGGAG
Cyp7a1-F	AGCAACTAAACAACCTGCCAGTACTA
Cyp7a1-R	GTCCGGATATTCAAGGATGCA
Cyp8b1-F	GGCTGGCTTCCTGAGCTTATT
Cyp8b1-R	ACTTCCTGAACAGCTCATCGG
Cyp7b1-F	TAGCCCTCTTTCCTCCACTCATA
Cyp7b1-R	GAACCGATCGAACCTAAATTCCT
Cyp27a1-F	GCCTCACCTATGGGATCTTCA
Cyp27a1-R	TCAAAGCCTGACGCAGATG
Mrp2-F	GGATGGTGACTGTGGGCTGAT
Mrp2-R	GGCTGTTCTCCCTTCTCATGG
Mrp3-F	TCCCACTTTTCGGAGACAGTAAC
Mrp3-R	ACTGAGGACCTTGAAGTCTTGGA
Hmgcs1-F	GTGGCACCGGATGTCTTTG
Hmgcs1-R	ACTCTGACCAGATACCACGTT
Hmgcr-F	AGCTTGCCCGAATTGTATGTG
Hmgcr-R	TCTGTTGTGAACCATGTGACTTC
Npc1l1-F	GCTTCTTCCGCAAGATATACACTCCC
Npc1l1-R	GAGGATGCAGCAATAGCCACATAAGAC
Abcg5-F	TCTCCGCGTCCAGAACAAC
Abcg5-R	CATTGAGCATGCCGGTGTAT

Abcg8-F	GACAGCTTCACAGCCCACAA
Abcg8-R	GCCTGAAGATGTCAGAGCGA
Acc1-F	GCCTCTTCCTGACAAACGAG
Acc1-R	TGACTGCCGAAACATCTCTG
Fas1-F	TGGTGAATTGTCTCCGAAAAGA
Fas1-R	CACGTTCATCACGAGGTCATG
Scd1-F	GCGATACACTCTGGTGCTCA
Scd1-R	CCCAGGGAAACCAGGATATT
Srebf1-F	TAGAGCATATCCCCCAGGTG
Srebf1-R	GGTACGGGCCACAAGAAGTA
Fgfr4-F	GCCTCCGACAAGGATTTGGCA
Fgfr4-R	GAGTGCAGACACCCAGCAGGT
Ldlr-F	CGCGGATCTGATGCGTCGCT
Ldlr-R	CGGCCCTGGCAGTTCTGTGG
Lxr-F	GCGTCCATTCAGAGCAAGTGT
Lxr-R	TCACTCGTGGACATCCCAGAT
Srebf2-F	GTGGAGCAGTCTCAACGTCA
Srebf2-R	TGGTAGGTCTCACCCAGGAG
Abca1-F	GGTTTGGAGATGGTTATACAATAGTTGT
Abca1-R	CCCGGAAACGCAAGTCC
Abcg1-F	TCACCCAGTTCTGCATCCTCTT
Abcg1-R	GCAGATGTGTCAGGACCGAGT
Fxr-F	TGTGAGGGCTGCAAAGGTTT
Fxr-R	ACATCCCCATCTCTCTGCAC

Shp-F	CGATCCTCTTCAACCCAGATG
Shp-R	AGGGCTCCAAGACTTCACACA
Fgf15-F	GAGGACCAAAACGAACGAAATT
Fgf15-R	ACGTCCTTGATGGCAATCG
Ibat-F	ACCACTTGCTCCACACTGCTT
Ibat-R	CGTTCCTGAGTCAACCCACAT
Ibabp-F	CAGGAGACGTGATTGAAAGGG
Ibabp-R	GCCCCCAGAGTAAGACTGGG
Osta-F	TGTTCCAGGTGCTTGTCATCC
Osta-R	CCACTGTTAGCCAAGATGGAGAA
Ostb-F	GATGCGGCTCCTTGGAATTA
Ostb-R	GGAGGAACATGCTTGTCATGAC
IL-1β-F	TGTAATGAAAGACGGCACACC
IL-1β-R	TCTTCTTTGGGTATTGCTTGG
IL-6-F	CCACTTCACAAGTCGGAGGCTTA
IL-6-R	GCAAGTGCATCATCGTTGTTCATAC
TNF-α-F	ATGAGCACAGAAAGCATGATC
TNF-α-R	TACAGGCTTGTCACTCGAATT
